# Evaluation of the Metabochip Genotyping Array in African Americans and Implications for Fine Mapping of GWAS-Identified Loci: The PAGE Study

**DOI:** 10.1371/journal.pone.0035651

**Published:** 2012-04-23

**Authors:** Steven Buyske, Ying Wu, Cara L. Carty, Iona Cheng, Themistocles L. Assimes, Logan Dumitrescu, Lucia A. Hindorff, Sabrina Mitchell, Jose Luis Ambite, Eric Boerwinkle, Petra Buzkova, Chris S. Carlson, Barbara Cochran, David Duggan, Charles B. Eaton, Megan D. Fesinmeyer, Nora Franceschini, Jeffrey Haessler, Nancy Jenny, Hyun Min Kang, Charles Kooperberg, Yi Lin, Loic Le Marchand, Tara C. Matise, Jennifer G. Robinson, Carlos Rodriguez, Fredrick R. Schumacher, Benjamin F. Voight, Alicia Young, Teri A. Manolio, Karen L. Mohlke, Christopher A. Haiman, Ulrike Peters, Dana C. Crawford, Kari E. North

**Affiliations:** 1 Department of Statistics and Biostatistics, Rutgers University, Piscataway, New Jersey, United States of America; 2 Department of Genetics, University of North Carolina, Chapel Hill, North Carolina, United States of America; 3 Division of Public Health Sciences, Fred Hutchinson Cancer Research Center, Seattle, Washington, United States of America; 4 University of Hawaii Cancer Center, Honolulu, Hawaii, United States of America; 5 Department of Medicine, Stanford University School of Medicine, Stanford, California, United States of America; 6 Center for Human Genetics Research, Vanderbilt University, Nashville, Tennessee, United States of America; 7 Office of Population Genomics, National Human Genome Research Institute, National Institutes of Health, Bethesda, Maryland, United States of America; 8 Department of Molecular Physiology and Biophysics, Vanderbilt University, Nashville, Tennessee, United States of America; 9 Information Sciences Institute, University of Southern California, Los Angeles, California, United States of America; 10 Human Genetics Center, University of Texas Health Science Center, Houston, Texas, United States of America; 11 Department of Biostatistics, University of Washington, Seattle, Washington, United States of America; 12 Sponsored Programs, Baylor College of Medicine, Houston, Texas, United States of America; 13 Translational Genomics Research Institute, Phoenix, Arizona, United States of America; 14 Center for Primary Care and Prevention, Department of Family Medicine and Epidemiology, Alpert Medical School of Brown University, Providence, Rhode Island, United States of America; 15 Department of Epidemiology, University of North Carolina, Chapel Hill, North Carolina, United States of America; 16 Department of Pathology, University of Vermont, Burlington, Vermont, United States of America; 17 Department of Biostatistics, University of Michigan, Ann Arbor, Michigan, United States of America; 18 Department of Genetics, Rutgers University, Piscataway, New Jersey, United States of America; 19 Department of Epidemiology and Medicine, University of Iowa, Iowa City, Iowa, United States of America; 20 Division of Public Health Sciences, Wake Forest University School of Medicine, Winston-Salem, North Carolina, United States of America; 21 Keck School of Medicine, University of Southern California, Los Angeles, California, United States of America; 22 Medical and Population Genetics, Broad Institute of Harvard and MIT, Cambridge, Massachusetts, United States of America; 23 Carolina Center for Genome Sciences, University of North Carolina, Chapel Hill, North Carolina, United States of America; Peninsula College of Medicine and Dentistry, United Kingdom

## Abstract

The Metabochip is a custom genotyping array designed for replication and fine mapping of metabolic, cardiovascular, and anthropometric trait loci and includes low frequency variation content identified from the 1000 Genomes Project. It has 196,725 SNPs concentrated in 257 genomic regions. We evaluated the Metabochip in 5,863 African Americans; 89% of all SNPs passed rigorous quality control with a call rate of 99.9%. Two examples illustrate the value of fine mapping with the Metabochip in African-ancestry populations. At *CELSR2/PSRC1/SORT1*, we found the strongest associated SNP for LDL-C to be rs12740374 (*p* = 3.5×10^−11^), a SNP indistinguishable from multiple SNPs in European ancestry samples due to high correlation. Its distinct signal supports functional studies elsewhere suggesting a causal role in LDL-C. At *CETP* we found rs17231520, with risk allele frequency 0.07 in African Americans, to be associated with HDL-C (*p* = 7.2×10^−36^). This variant is very rare in Europeans and not tagged in common GWAS arrays, but was identified as associated with HDL-C in African Americans in a single-gene study. Our results, one narrowing the risk interval and the other revealing an associated variant not found in Europeans, demonstrate the advantages of high-density genotyping of common and rare variation for fine mapping of trait loci in African American samples.

## Introduction

A half-decade of successes from Genome-Wide Association Studies (GWAS) have identified more than 1,200 SNPs associated with 210 complex human traits [1]. While GWA studies have produced novel genotype-phenotype associations, such studies are but initial steps in the process of understanding the biology relevant to human disease. Post-GWAS studies are necessary to identify risk or causal variants given that most GWAS findings reflect common variants, targeted for genotyping, that presumably are in linkage disequilibrium with the actual causal variants.

A common post-GWA study design involves fine mapping a region of interest with dense genotyping or sequencing. Fine mapping in diverse populations has the advantage that linkage disequilibrium architectures vary across populations, potentially narrowing the region of interest containing the putative causal variant [2], [3]. Fine mapping in an alternate population can also identify additional causal variants that are absent or rare in the discovery population.

Custom genotyping arrays such as the Metabochip, an array based on the Illumina iSelect platform, represent an attractive approach for multi-locus fine mapping. Containing 196,725 SNPs, including content on less common variation from the 1000 Genomes Project [4], the Metabochip was developed for replication and fine mapping of susceptibility loci associated with one or more metabolic and cardiovascular traits. It costs substantially less than leading GWAS arrays or sequencing technologies but enables analysis of low-frequency variants at fine mapped loci. Sanna *et al* recently explored its use, along with sequencing, to increase the explained heritability of LDL-C in seven genes, including *SORT1*, in a Sardinian sample [5]. Although the Metabochip is specifically designed for metabolic and cardiovascular trait association, similar custom arrays targeting cancer and immune disease-related phenotypes are in early use [6], [7]. The performance of such arrays in non-European-descent populations is not known, although the use of these custom arrays in ancestrally diverse populations allows for the potential narrowing of risk intervals in susceptibility loci uncovered by GWAS, the discovery of low frequency susceptibility variants, and the identification of additional independent signals in known loci.

The “Population Architecture using Genomics and Epidemiology” (PAGE) study [8] recently undertook a pilot study with the Metabochip in African Americans to facilitate fine mapping around key GWAS loci, addressing the major goal of PAGE, the assessment of how GWAS-identified variants generalize across populations. In the present work, we describe the performance of the Metabochip in African American individuals from the PAGE study. We give two examples of the benefit this array in an African American sample by fine mapping two established loci for serum levels of lipids, namely the low-density lipoprotein cholesterol (LDL-C) locus at *CELSR2/PSRC1/SORT1* and the high-density lipoprotein cholesterol (HDL-C) locus at *CETP* using the Metabochip in this African American population. The examples illustrate two types of results that can be expected by fine mapping in African Americans: narrowing of the risk interval and discovery of a population-specific variant. On the basis of the results presented here PAGE has decided to use the Metabochip as its primary genotyping platform for the immediate future.

## Results

### Quality Control

We genotyped a total of 6,241 African American individuals previously recruited to 3 cohort studies: the Atherosclerosis Risk in Communities Study (ARIC) [9], the Multiethnic Cohort (MEC) [10], and the Women's Health Initiative (WHI) [11]. We removed 0.9% of the samples for sample QC reasons and 5.2% of the samples as representing half of a first-degree relative pair, leaving 5,863 unique individuals. Details are shown in Supporting Information Table S1.

Before SNP quality control, the overall call rates were 98.98%, 97.74%, and 97.70% for ARIC, MEC, and WHI, respectively. We summarize the SNP QC results in Supporting Information Table S2. We considered a total of 14,328 (7.3%) variants as technical failures because of the GenCall or cluster separation score, call rate, Mendelian error rate, replication error rate, or deviation from Hardy Weinberg equilibrium. There were an additional 5,248 (2.7%) SNPs that were failed, despite passing technical QC, because the probe sequence matched poorly to the reference genome (no or more than one match found in reference; probe sequence differed from reference; neither SNP allele matched reference; a second, equally good, match to a random contig, in addition to stated position; one or more second-best hits in the reference that differ only by a single base). We found that the discordance between calls by GenCall and GenoSNP, an alternative sample-based genotype calling program [12], was low (0.1% of genotypes; 0.8% of SNPs exceeded the QC threshold for discordance, but 1.2% of SNPs with MAF less than 0.01 did so), as was the discordance in YRI samples with HapMap database genotypes for the 105,722 SNPs in common (0.3% of genotypes; 1.1% of overlapping SNPs exceeded the QC threshold for discordance). SNPs with excessive discordance of either type were failed (Supporting Information Table S3). An additional 6.8% of SNPs were monomorphic across all three PAGE cohorts. Overall, 161,098 (81.9%) SNPs on the Metabochip passed our quality control criteria and were polymorphic. The call rate for SNPs passing QC was 99.9%. The QC profile of SNPs identified via the 1000 Genomes Project did not appreciably differ from other SNPs with one exception: 99.4% of the monomorphic SNPs were from the 1000 Genomes Project.

### Characteristics and Distribution of Allele Frequencies

**Figure 1:**
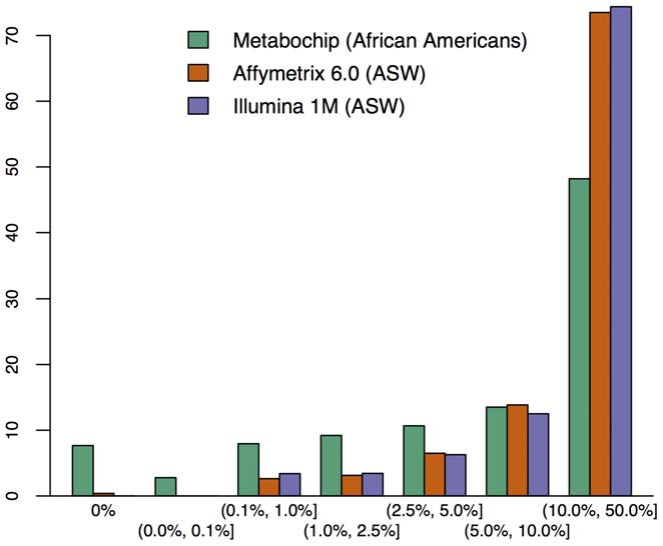
Distribution of minor allele frequencies on the Metabochip, the Affymetrix 6.0, and the Illumina 1 M. Intervals are open on the left and closed on the right. The Metabochip MAFs are calculated from PAGE African American samples, while the Affymetrix and Illumina GWAS array MAFs are drawn from the HapMap ASW (African ancestry in Southwest USA) population, a difference not expected to substantially impact the allele frequency distribution.

**Figure 2:**
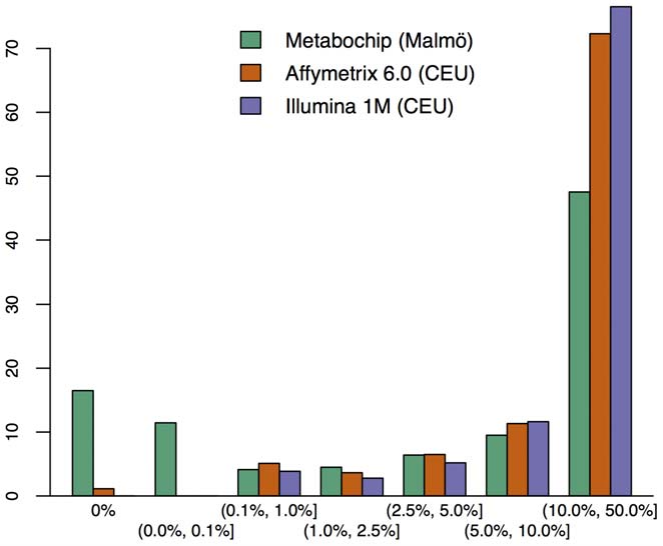
Distribution of minor allele frequencies on the Metabochip, the Affymetrix 6.0, and the Illumina 1 M. Same comparison as in Figure 1, except that in this figure the minor allele frequencies on the Metabochip are determined from the Malmö, Sweden, sample while those on the Affymetrix 6.0 and Illumina 1 M GWAS arrays are determined from the HapMap CEU sample.

The Metabochip differs from most GWAS arrays in its coverage of rare and low frequency variants. Figure 1 shows the percentage of SNPs at various minor allele frequencies (MAFs) for the African American PAGE study samples genotyped on the Metabochip compared to HapMap ASW samples genotyped on the Affymetrix 6.0 and the Illumina 1 M arrays. A total of 21.6% of the polymorphic Metabochip SNPs have MAF less than 0.025, compared to 5.8% and 6.8% for the Affymetrix 6.0 and Illumina 1 M arrays, respectively. Differences between frequencies in the Metabochip and GWAS chips are also notable in European and European-descent populations (Figure 2) particularly for SNPs with MAF of less than or equal to 0.001. The proportion of SNPs passing QC across MAFs ranged from 85% to 92% with no trend by MAF (Supporting Information Table S4).

### Fine mapping in Metabochip: LDL-C case study

**Figure 3:**
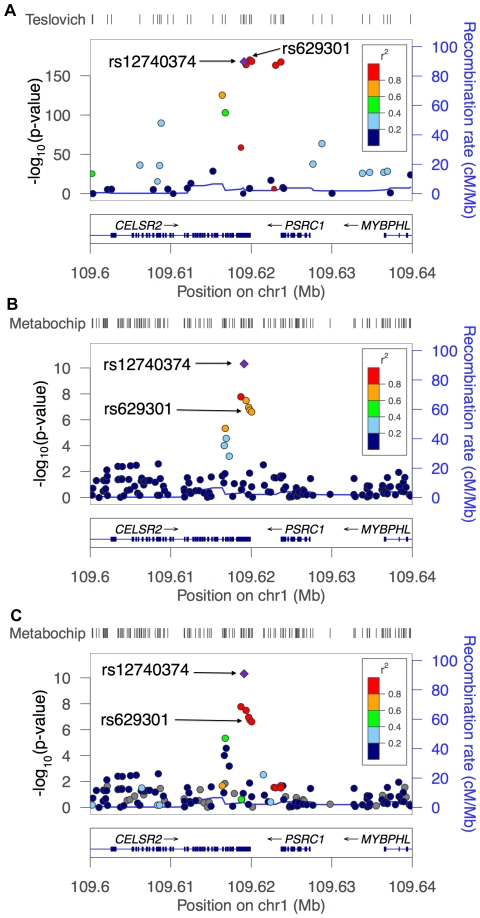
Regional association plots for LDL-C near the *CELSR2/PSRC1/SORT1* locus. The LDL-C association signal near the *CELSR2/PSRC1/SORT1* locus is narrower in African Americans than Europeans. (A) Association results from European-ancestry samples [13] colored by LD (*r^2^*) in CEU with rs12740374. (B) Association results in African Americans from PAGE colored by LD (*r^2^*) in the same sample with rs12740374; (C) The same association results as in (B) but colored by their correlation (*r^2^*) in the Malmö, Sweden, sample [32] with rs12740374. Positions shown are based on hg18. Recombination rate shown is based jointly on the CEU, YRI and JPT+CHB HapMap samples [38].

As an illustration of the advantages of the Metabochip in fine mapping signals from European-descent GWAS using African-descent individuals, we compared the region of our most significant association with LDL-C in the PAGE African American participants with the same region in a GWAS meta-analysis of lipid associations in over 100,000 individuals of European ancestry [13]. (A comprehensive analysis of lipids associations from the Metabochip in this sample and others is described in Wu *et al*, in preparation.) In this region, as well as the region discussed below, most of the Metabochip SNPs are poorly tagged by a GWAS array; in YRI, more than 80% have a maximum *r* ^2^ less than 0.5 with SNPs on the Illumina 1 M array (Table 1). Our most significant result for LDL-C was at SNP rs12740374 on chromosome 1p13 at the *CELSR2/PSRC1/SORT1* locus, with a *p*-value of 3.48×10^−11^. The estimated effect at this locus is a mean increase, for each A allele, of 6.5 mg/dl of LDL-C (95% CI: 4.6 to 8.5) with a heterogeneity *I^2^* of 31% (considered low to moderate [14]) and mean allele frequency of 0.25. Association of this SNP with LDL-C was identified previously in European-ancestry individuals [15] and in African American individuals [16]. (The latter study included most of the ARIC sample analyzed here.) In this region, Teslovich and colleagues [13] reported the strongest LDL-C association at the nearby SNP rs629301 with an effect size of 5.6 mg/dl. Figure 3A shows the Teslovich *et al* results in this region.

**Table 1:** Coverage of 2 Metabochip regions by SNPs on the Illumina 1 M GWAS array.

Maximum r^2^ with Illumina 1 M array SNP	*CELSR2/PSRC1/SORT1*	*CETP*
[0, 0.50]	105	76
(0.50, 0.99]	11	2
(0.99, 1.00]	7	4
Illumina 1 M array SNPs on Metabochip	20	16
Total	143	98

**Figure 4:**
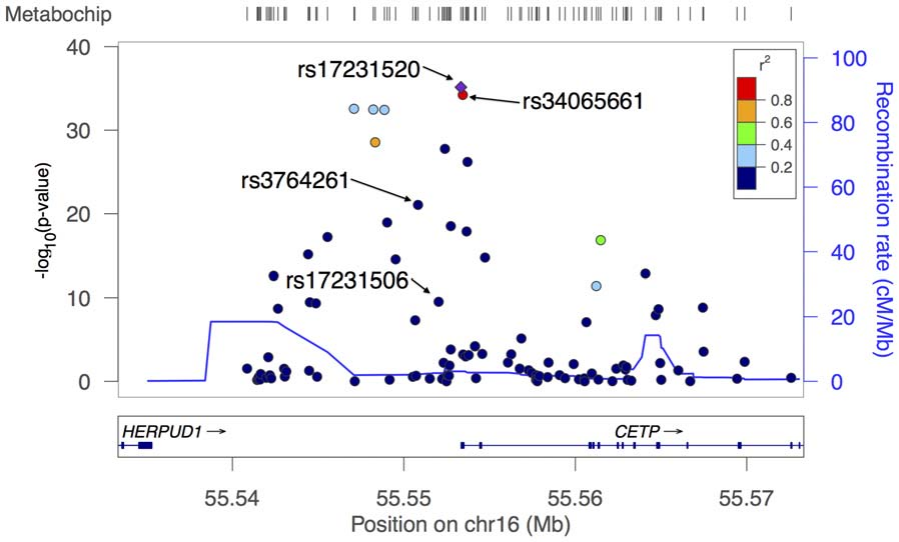
Regional association plot for HDL-C results from PAGE in African Americans near the *CETP* locus. SNPs are colored by their LD (*r^2^*) in the same sample with rs17231520.

Linkage disequilibrium was calculated in ASW (African ancestry in Southwest USA). The *CELSR2/PSRC1/SORT1* region refers here to the 40 kb region shown in Figure 3. The *CETP* region here refers to the 30 kb region shown in Figure 4. Counts in the table refer to polymorphic SNPs on the Metabochip that passed quality control.

Figure 3B demonstrates the pattern of association in this region using the PAGE Metabochip results and showing the strength of LD in the African American sample relative to our most significant SNP, rs12740374. For comparison, the same results are re-plotted in Figure 3C with LD shown from a sample of European-descent individuals based in Malmö, Sweden. Of note, with the Malmö LD there are eight SNPs that have an *r* ^2^>0.9 with the rs12740374 index SNP. Although rs629301 did not pass QC in the Malmö study, the 1000 Genomes Project [4] data shows that rs629301 and rs12740374 have *r* ^2^ = 1.0 in CEU. In contrast, when examining the LD based on the PAGE African American sample, there was only one SNP (rs7528419) with an *r* ^2^ value greater than 0.9 with rs12740374. The next five most correlated SNPs, including rs629301, had *r^2^* values between 0.60 and 0.62.

### Fine mapping in Metabochip: HDL-C case study

A second illustration of the use of the Metabochip in fine mapping is at *CETP* on chromosome 16q21 (Figure 4), the location of our most significant SNP associations for HDL-C in the PAGE African American samples at rs17231520 (*p* = 7.2×10^−36^) and rs34065661 (Ala15Gly, *p* = 6.0×10^−35^). Thompson *et al* [17] found rs17231520 to be associated with HDL-C in African Americans in a study of SNPs in *CETP*. The estimated effects at these SNPs were 7.81 mg/dl (95% CI: 6.58 to 9.03) per A allele and 7.71 mg/dl (95% CI: 6.48 to 8.93) per G allele for rs17231520 and rs34065661, respectively, with a heterogeneity *I^2^* of 0% for both. These SNPs showed stronger estimated effects with HDL-C levels in the PAGE study than the GWAS index SNP rs3764261 did in the European-descent based Global Lipids Genetic Consortium meta-analysis [13]. From the PAGE sample we estimated an effect size of 3.3 (2.64, 4.00) mg/dl for each A allele in rs3764261 (*p* = 8.7×10^−22^) and an allele frequency of 0.32, compared to 3.4 and 0.32, respectively, given by the Global Lipids Genetic Consortium. In the PAGE African American sample the two lead SNPs, rs17231520 and rs34065661, were in strong LD with each other (*r* ^2^ = 0.998), but both were only weakly correlated with rs3764261 (*r* ^2^ = 0.16). The MAFs at rs17231520 and rs34065661 were 0.07 for both variants in African Americans, but the SNPs were very rare or monomorphic in Europeans and Asians (Malmö, Sweden sample: 0.0007 for rs17231520 and 0.0002 for rs34065661; 1000 Genomes Project EUR: MAF = 0 for rs17231520 and MAF = 0.002 for rs34065661; 1000 Genomes Project ASN: MAF = 0 for both SNPs). When conditioned on rs17231520, the strongest remaining HDL-C-associated SNP in PAGE was rs17231506 (*p* = 4.0×10^−15^ with conditioning while *p* = 3.2×10^−10^ without conditioning), which showed moderate LD with the GWAS index SNP rs3764261 (*r* ^2^ = 0.372, *p* for rs17231506 conditional on rs3764261 = 0.79). Although an argument for independence via a conditioning analysis is not at all definitive [18], these data are consistent with the presence of two independent susceptibility signals for HDL-C at this locus in African Americans, one of which (rs17231506/rs3764261) is shared with European Americans.

## Discussion

Published GWA studies have been predominantly conducted in European-descent populations [2]; the degree to which GWAS arrays (along with imputation using HapMap or 1000 Genomes data) will adequately capture common genetic variants that influence complex traits in ancestrally diverse populations remains to be fully determined. The difficulty in generalizing results from European-descent GWAS to other populations, particularly African Americans, is consistent with the belief that SNPs on GWAS arrays do not generally represent causal or functional SNPs. Because LD patterns differ across populations, tag SNPs genotyped in populations of non-European ancestry may not capture the associations observed in European-ancestry populations. Genotyping with large-scale custom array platforms like the Metabochip provides an opportunity 1) to test for generalization of previous GWAS identified SNPs across ancestrally diverse populations, 2) to fine map the loci, and 3) to possibly identify additional variants that independently associate with these traits [19].

By design, the Metabochip provides a greater depth of coverage, both for overall SNP density and for rare SNPs, in specific targeted regions of the genome than the Affymetrix 6.0 or Illumina 1 M arrays. This greater depth of coverage is evident in both the Malmö European and PAGE African American populations. The Metabochip performed well on QC measures in the PAGE African American sample with 85% to 89% of SNPs passing QC across the range of MAFs and overall call rates above 97%.

To demonstrate the utility of the fine mapping approach, we assessed two well-known loci. At the *CELSR2/PSRC1/SORT1* locus on chromosome 1, Teslovich and colleagues have reported a strong association of LDL-C with rs629301 and several nearby SNPs [13]. In contrast, Lettre and colleagues [16] demonstrated the strongest association in African-descent populations of LDL-C in this locus with rs12740374, consistent with our findings. Musunuru and colleagues [20] conducted a series of human *in vitro* and *in vivo* experiments demonstrating that this common noncoding variant rs12740374 creates a transcription factor binding site that alters the hepatic expression of *SORT1*, thereby identifying a functional variant at the *CELSR2/PSRC1/SORT1* locus. Figure 3A demonstrates that several nearby SNPs are both highly significant and in high LD in European-descent populations. However, because of this high LD, it is difficult to discern if any of these SNPs is a true causal variant. Figure 3B, which includes additional data on fine-mapping SNPs in an African American sample, suggests that in these subjects a true causal variant can be pinpointed to a region in high LD with rs12740374. Thus, the combination of locally high SNP density on the Metabochip and the less extensive LD in African Americans allowed us to distinguish rs12740374 from other SNPs that are in LD in Europeans. These study findings illustrate how fine mapping in a African Americans can refine a GWAS signal and, in this case, uncover a putative functional variant.

Similarly, our findings at the *CETP* locus on chromosome 16, associated with HDL-C, demonstrate the value of high-density genotyping to identify putatively functional alleles that are not present on GWA arrays. In this case, rs17231520, which showed the most significant association in this region in the PAGE sample, is located in the *CETP* promoter or 5′ UTR of different isoforms of the gene [21]. This variant, described previously to be associated with HDL-C in African Americans [17], resides in an SP1/SP3 binding site and has been shown to influence gene transcriptional activity and CETP protein function. Although Polyphen-2 [22] predicts that the non-synonymous SNP rs34065661 (Ala15Gly), our second most significantly associated SNP at this locus, would have minimal adverse consequences on protein structure and function, this structural change may have a biological effect (and, of course, prediction tools are imperfect). As one of the residues forming the signal peptide that directs transport of the CETP protein into the lipid membrane, the variant could affect CETP localization. Neither rs17231520 not rs34065661 is present on the Affymetrix 6.0 or Illumina 1 M arrays, nor are any proxies with *r* ^2^>0.65 according to the proxy-SNP identification tool SNAP [23] (in either CEU and YRI based on 1000 Genomes Pilot 1 data; the SNPs are not in HapMap 3 Release 2). The two SNPs can, however, be moderately well imputed from 1000 Genomes Phase I v2 data (Rsq = 0.79 for both), suggesting that even in African Americans these lead associations would be found by a GWAS at best only via imputation. Taken together, our findings suggest that the high-density genotyping in the Metabochip allowed detection at an already established locus of a presumably functional SNP that appears to be specific to recent African descent populations, given MAFs of 0.07 in African Americans but near 0 in Europeans and Asians. Further work may identify additional functional variants at *CETP* and their relative contributions in other populations.

An alternative to genotyping with the Metabochip would be to impute genotypes determined using a GWAS array and a 1000 Genomes reference panel [24]. Doing so requires GWAS array genotypes, of course, and concerns have been raised about the sensitivity of association results in imputed rare alleles [25]. An advantage would be that the imputed SNPs would not be limited to selected regions on the genome. Imputation from GWAS genotypes to Metabochip SNP genotypes is discussed in Liu *et al* [26].

In summary, our results demonstrate the feasibility of using the Metabochip and the content revealed in the 1000 Genomes Project to generalize GWAS signals initially discovered in European-descent populations to African Americans from multiple sites in the PAGE study. We expect this custom genotyping approach to point to additional underlying functional variants once it is applied to a diverse set of populations. Future studies are expected to incorporate a combination of approaches, including GWAS and sequencing, to explain the heritable component of complex traits. In the meantime, the wide-scale application of targeted custom genotyping strategies such as the Metabochip is clearly warranted and cost-effective.

## Materials and Methods

### Study Populations

The PAGE study consists of four study sites and a Coordinating Center [8]. The four study sites comprise large, diverse population-based studies: the Epidemiologic Architecture for Genes Linked to Environment (EAGLE), which accesses three National Health and Nutrition Examination Surveys; the Multiethnic Cohort (MEC); the Women's Health Initiative (WHI); and Causal Variants Across Life Course (CALiCo), a collection of cardiovascular cohorts including the Atherosclerosis Risk in Communities Study (ARIC). The PAGE Metabochip Pilot Study was limited to African American participants from ARIC [9], n = 3,663, MEC [10], n = 458, and WHI [11], n = 2,120, for a total of 6,241 individuals.

### Brief Description of the Metabochip Content

The design of the Metabochip is fully described by Voight *et al* (in preparation). Approximately 33% of the Metabochip SNPs consist of replication targets and 62% are located in fine mapping regions; the other 5% serve a variety of purposes such as CNV tagging, gender assessment, and coverage of the HLA region. SNPs identified in the 1000 Genomes Project Pilot 1 [4] make up about 60% of the Metabochip SNPs. The fine mapping targets focus on loci that have achieved genome-wide significance for one or more metabolic and atherosclerotic-cardiovascular traits, including type 2 diabetes, fasting glucose and insulin, coronary artery disease/myocardial infarction, LDL-C, HDL-C, triglycerides, body mass index, waist-to-hip ratio, systolic blood pressure, diastolic blood pressure, and QT interval. The density of SNPs in the fine mapping regions varies according to the priorities of the Metabochip design group with a median density of one SNP per 370 bases in these regions. A total of 257 loci were selected for fine mapping with the surrounding regions covering a total of 45.52 Mb after accounting for overlaps (14.17 Mb for the densest fine mapping regions). More details can be found at http://www.sph.umich.edu/csg/kang/MetaboChip/

### Genotyping and Quality Control Assessment

We genotyped samples at the Human Genetics Center of the University of Texas-Houston, the University of Southern California Genomics Core, and the Translational Genomics Research Institute (TGen) for the ARIC, MEC, and WHI cohorts, respectively. Each center followed the manufacturer's recommendation (Illumina, Inc.) and at a minimum assessed standard quality controls procedures (e.g., intra- and inter-batch controls, blinded duplicates) as well as those provided by the manufacturer. Each center also genotyped 90 HapMap YRI (Yoruba in Ibadan, Nigeria) samples to facilitate cross-study quality control (QC). To reduce possible batch effects caused by separate genotyping centers, we called genotypes separately for each study at the PAGE Coordinating Center under a common protocol, using GenomeStudio with the GenCall 2.0 algorithm (Illumina Inc., San Diego, CA). We also called all genotypes with the GenoSNP genotype-calling algorithm [12], given that the Metabochip includes SNPs with much lower minor allele frequencies (MAFs) than are usually called with GenCall. The GenoSNP algorithm may be more successful for rare genotypes because it is sample-based, rather than SNP-based [27]. Genotypes called by GenCall were used in analyses, but discordance between the two methods was used as a QC filter. The QC criteria, including thresholds, for SNPs are described in Supporting Information Table S3. QC statistics were calculated using GenomeStudio and PLINK [28].

We excluded samples with a call rate below 0.95 or an inbreeding coefficient (F) above 0.15 from further analysis [29]. Prior to quantitative genetic analyses, we identified related persons using PLINK by estimating identical-by-descent (IBD) statistics for all pairs. When apparent first-degree relative pairs were identified, we selected one individual from each pair by excluding the relative with a lower call rate from further analysis. We determined principal components of ancestry using EIGENSOFT [30], [31] and excluded apparent ancestral outliers from further analysis as described in the Supporting Information S1.

### LD Estimates and Statistical Analysis of Association of Lipid Levels

To aid in the interpretation of fine mapping results, we calculated the *r* ^2^ linkage disequilibrium statistic in our African American PAGE Metabochip Pilot Study sample in 500 kb sliding windows using PLINK. In addition, the Malmö Diet and Cancer Study [32] provided LD and frequency information (but not individual-level information or any trait information) on 2,143 control subjects from a Swedish population. We also obtained allele frequencies from HapMap [33] for ASW (African ancestry in Southwest USA) and CEU (Northern and Western European ancestry) samples.

We measured serum HDL-C using standard enzymatic methods and calculated fasting levels of LDL-C using the Friedewald equation [34], assigning missing values of LDL-C to individuals with triglyceride levels greater than 400 mg/dl. As fewer than 10% of these participants were on lipid-lowering medications at the time of blood draw, we did not exclude individuals on such medications in these analyses; we did not attempt to adjust lipid levels for medicated individuals. In a previous PAGE study, exclusion of lipid-medicated participants in the ARIC, MEC, and WHI cohorts did not appreciably alter lipids association results [35]. We used standard linear regression models with the first two principal components of ancestry for a global ancestry adjustment and with age and sex as covariates. We encoded SNPs using the linear additive model and combined association results across cohorts using an inverse variance fixed effect meta-analysis approach implemented in METAL [36]. We also accessed publicly available data from the Global Lipids Genetic Consortium [13] at http://www.sph.umich.edu/csg/abecasis/public/lipids2010/ for comparison purposes. We used LocusZoom [37] for association plots.

## Supporting Information

Supporting Information S1 Additional Methods.
(DOC)

Table S1 Number of samples genotyped. “Failed QC” includes ancestry outliers. “First degree relative pairs” refers to apparent first-degree relative pairs based on genotypes; one member of each pair was retained for the analyses.
(DOCX)

Table S2 Quality control outcomes by SNP. “Technical Quality Control Failures” refers to SNPs failing steps 1–7 in Supporting Information Table S3.
(DOCX)

Table S3 SNP quality control criteria. GenTrain and cluster separation scores are Illumina-provided genotype metrics. SNPs that failed only for GenTrain or cluster separation scores at only one site and passed a manual inspection were classified as passing. Otherwise, SNPs failing any criterion for any one study were classified as failed across PAGE.
(DOCX)

Table S4 Metabochip SNP overall pass rate by minor allele frequency in the PAGE African American sample. Intervals are open on the left and closed on the right. The overall pass rate, including monomorphic SNPs, was 88.7%.
(DOCX)
