# Supplementary material for: Evaluation of the Metabochip Genotyping Array in African Americans and Implications for Fine Mapping of GWAS-Identified Loci: The PAGE Study
Source: PLoS One. 2012 Apr 23;7(4):e35651. doi: 10.1371/journal.pone.0035651 (PMC3335090; doi:10.1371/journal.pone.0035651)
Supplement: Supporting Information S1 — Additional Methods. (DOC) [file pone.0035651.s001.doc]

**Supporting Information S1:**

**Additional Methods**

**1. Descriptions of Participants, Data Collection, and Genotyping by Study:**

**Causal Variants Across the Life Course (CALiCo).**

CALiCo is a consortium of six demographically diverse population based studies and a central laboratory. This network contributes a maximum of approximately 58,000 men and women ranging in age from childhood to older adulthood. Only one CALiCo study has Metabochip data, the **Atherosclerosis Risk in Communities (ARIC) Study.** The ARIC study is a multi-center prospective investigation of atherosclerotic disease in a predominantly bi-racial population. White and African American men and women aged 45-64 years at baseline were recruited from four communities: Forsyth County, North Carolina; Jackson, Mississippi; suburban areas of Minneapolis, Minnesota; and Washington County, Maryland[1]. A total of 15,792 individuals participated in the baseline examination in 1987-1989, with follow-up examinations in approximate 3-year intervals, during 1990-1992, 1993-1995, and 1996-1998. After the institutional review board at every participating university approved the ARIC Study protocol, written informed consent was obtained from each participant.

*Data Collection:* Race and lipid-lowering medication use were self-reported. Fasting blood was drawn while the participant was seated from an antecubital vein into tubes containing EDTA, and plasma was obtained by centrifugation at 4°C and stored at −70°C until analysis. All samples were sent to the ARIC Central Lipid Laboratory for processing. Triglycerides were determined by enzymatic methods[2] using the Cobas Bioanalyzer (Roche). The Friedewald equation was used to calculate LDL-C in those with triglyceride levels under 400 mg/dl[3]. Blood chemistries were performed at the Central Chemistry Laboratory of the University of Minnesota, and blood lipid analyses were performed at the University of Texas, Houston.

**Multiethnic Cohort (MEC).**

The MEC is a population-based prospective cohort study consisting of 215,251 men and women, and comprises mainly five self-reported racial/ethnic populations: African Americans, Japanese Americans, Latinos, Native Hawaiians and European Americans[4]. The MEC was designed to provide prospective data on exposures and biomarkers potentially involved in cancer initiation and progression across groups with distinct cultural and dietary patterns. Between 1993 and 1996, adults between 45 and 75 years old were enrolled by completing a 26-page, self-administered questionnaire asking detailed information about dietary habits, demographic factors, level of education, personal behaviors, and history of prior medical conditions (e.g. diabetes). Between 1995 and 2004, blood specimens were collected from ~67,000 MEC participants at which time a short questionnaire was administered to update certain exposures, and collect current information about medication use. Study protocols and consent forms were approved by the institutional review boards at all participating institutions.

*Data Collection:* Medication use and fasting information was collected at the time of blood collections. LDL, HDL, and triglycerides concentrations were measured using standard clinical assays.

**Women’s Health Initiative (WHI).**

WHI is a long-term national health study that focuses on strategies for preventing heart disease, breast and colorectal cancer and fracture in postmenopausal women. A total of 161, 838 women aged 50–79 years old were recruited from 40 clinical centers in the US between 1993 and 1998[5]. WHI consists of an observational study, two clinical trials of postmenopausal hormone therapy (estrogen alone or estrogen plus progestin), a calcium and vitamin D supplement trial, and a dietary modification trial. Trial exclusion criteria have been described previously[6]. Study protocols and consent forms were approved by the institutional review boards at all participating institutions. A subset of 2151 African American WHI women with at least one baseline lipid measurement were selected.

*Data Collection:* Self-reported demographic, lifestyle and general health characteristics (current smoking, history of myocardial infarction, type 2 diabetes) were collected at baseline as described previously[6]. Baseline medication use (lipid-lowering and hormone replacement medications) was ascertained using a computer-driven medication inventory system at the first screening visit. Race/ethnicity was self-reported. Fasting LDL, HDL, and triglycerides concentrations were measured using standard clinical assays in a variety of core and ancillary WHI studies; measurements were normalized to correct for laboratory and study effect.

**2. Ancestry Estimation**

To determine ancestry, one member of each apparent second-degree relative pair was temporarily dropped. A subset of SNPs was selected for ancestry analysis by the following procedure: SNPS were first restricted to autosomal non-palindromic SNPs that passed QC and for which genotypes were available on the HapMap Phase 3 populations. SNPs in the HLA and 8p23-inversion regions were dropped and a low linkage disequilibrium subset, (pairwise LD < 0.2) selected to favor differences in frequency among the HapMap Phase 2 populations, was selected. EIGENSOFT was used to determine principal components[7,8]. Severe ancestry outliers were determined by analyzing the three samples’ genotypes along with HapMap genotypes in a joint analysis and then making a visual determination. Additional ancestry outliers were determined by EIGENSOFT’s default of exceeding 6 standard deviations in any of the first ten principal components. Principal components of ancestry were then determined separately for each study (excluding HapMap genotypes). The first ten components for each study were checked for association with the SNP list and SNPs with any p < 10^-8^ were dropped; new principal components were determined and the process was then repeated until no such SNPs remained. In this way no principal component of ancestry would be highly correlated with any one SNP, a potential cause of confounding in association studies. Ultimately, 23,602 SNPs were used to determine the principal components of ancestry.

References

1. The ARIC investigators (1989) The Atherosclerosis Risk in Communities (ARIC) Study: design and objectives. American Journal of Epidemiology 129: 687-702.

2. Nagele U, Hagele EO, Sauer G, Wiedemann E, Lehmann P, et al. (1984) Reagent for the enzymatic determination of serum total triglycerides with improved lipolytic efficiency. J Clin Chem Clin Biochem 22: 165-174.

3. Friedewald WT, Levy RI, Fredrickson DS (1972) Estimation of the concentration of low-density lipoprotein cholesterol in plasma, without use of the preparative ultracentrifuge. Clin Chem 18: 499-502.

4. Kolonel LN, Henderson BE, Hankin JH, Nomura AM, Wilkens LR, et al. (2000) A multiethnic cohort in Hawaii and Los Angeles: baseline characteristics. American Journal of Epidemiology 151: 346-357.

5. Anderson GL, Manson J, Wallace R, Lund B, Hall D, et al. (2003) Implementation of the women's health initiative study design. Annals of Epidemiology 13: S5-S17.

6. The Women's Health Initiative Study Group (1998) Design of the Women's Health Initiative clinical trial and observational study. Controlled Clinical Trials 19: 61-109.

7. Patterson N, Price AL, Reich D (2006) Population structure and eigenanalysis. PLoS Genetics 2: e190.

8. Price AL, Patterson NJ, Plenge RM, Weinblatt ME, Shadick NA, et al. (2006) Principal components analysis corrects for stratification in genome-wide association studies. Nature Genetics 38: 904-909.
