# Supplementary material for: Evaluation of the Metabochip Genotyping Array in African Americans and Implications for Fine Mapping of GWAS-Identified Loci: The PAGE Study
Source: PLoS One. 2012 Apr 23;7(4):e35651. doi: 10.1371/journal.pone.0035651 (PMC3335090; doi:10.1371/journal.pone.0035651)
Supplement: Table S1 — Number of samples genotyped. “Failed QC” includes ancestry outliers. “First degree relative pairs” refers to apparent first-degree relative pairs based on genotypes; one member of each pair was retained for the analyses. (DOCX) [file pone.0035651.s002.docx]

|  | ARIC | MEC | WHI | Total |
| --- | --- | --- | --- | --- |
| Individuals | 3663 | 458 | 2120 | 6241 |
| Failed QC | 27 | 1 | 27 | 55 |
| First-degree relative pairs | 306 | 2 | 15 | 323 |
| Included in analyses | 3330 | 455 | 2078 | 5863 |

**Supporting Information Table S1.** Number of samples genotyped. “Failed QC” includes ancestry outliers. “First degree relative pairs” refers to apparent first-degree relative pairs based on genotypes; one member of each pair was retained for the analyses.
