# Supplementary material for: Evaluation of the Metabochip Genotyping Array in African Americans and Implications for Fine Mapping of GWAS-Identified Loci: The PAGE Study
Source: PLoS One. 2012 Apr 23;7(4):e35651. doi: 10.1371/journal.pone.0035651 (PMC3335090; doi:10.1371/journal.pone.0035651)
Supplement: Table S2 — Quality control outcomes by SNP. “Technical Quality Control Failures” refers to SNPs failing steps 1–7 in Supporting Information Table S3. (DOCX) [file pone.0035651.s003.docx]

| Category | | N | Percentage |
| --- | --- | --- | --- |
|  | Total Number of SNPs | 196,725 | 100.0 |
|  | Technical Quality Control Failures | 14,328 | 7.3 |
|  | SNP Mapping problem | 5,248 | 2.7 |
|  | Discordant (GenoSNP) | 1,561 | 0.8 |
|  | Discordant (HapMap) | 1,111 | 0.6 |
|  | Monomorphic & passed QC | 13,379 | 6.8 |
|  | Polymorphic & passed QC | 161,098 | 81.9 |

**Supporting Information Table S2:** Quality control outcomes by SNP. “Technical Quality Control Failures” refers to SNPs failing steps 1–7 in Supporting Information Table S3.
