# Supplementary material for: Evaluation of the Metabochip Genotyping Array in African Americans and Implications for Fine Mapping of GWAS-Identified Loci: The PAGE Study
Source: PLoS One. 2012 Apr 23;7(4):e35651. doi: 10.1371/journal.pone.0035651 (PMC3335090; doi:10.1371/journal.pone.0035651)
Supplement: Table S3 — SNP quality control criteria. GenTrain and cluster separation scores are Illumina-provided genotype metrics. SNPs that failed only for GenTrain or cluster separation scores at only one site and passed a manual inspection were classified as passing. Otherwise, SNPs failing any criterion for any one study were classified as failed across PAGE. (DOCX) [file pone.0035651.s004.docx]

| Step  Number | Criteria |  | SNP Failure Determination |
| --- | --- | --- | --- |
| 1 | Illumina GenTrain Score |  | < 0.6 |
| 2 | Illumina Cluster Separation Score |  | < 0.4 |
| 3 | Call Rate |  | < 0.95 |
| 4 | Mendelian Errors in YRI |  | > 1 (out of 30 trios) |
| 5 | Replication Errors |  | > 2 |
| 6 | Hardy-Weinberg Equilibrium p-value |  | < 1 × 10^-6^ |
| 7 | Discordant calls from GWAS arrays on overlapping samples |  | > 10% |
| 8 | Discordant calls on YRI: across studies |  | > 3 (out of 90 samples) |
| 9 | Discordant calls from GenoSNP |  | > 3.3% |
| 10 | Discordant calls on YRI:PAGE consensus versus HapMap database |  | > 3 (out of 90 samples) |
|  |  |  |  |

**Supporting Information Table S3:** SNP quality control criteria. GenTrain and cluster separation scores are Illumina-provided genotype metrics. SNPs that failed only for GenTrain or cluster separation scores at only one site and passed a manual inspection were classified as passing. Otherwise, SNPs failing any criterion for any one study were classified as failed across PAGE.
