# Supplementary material for: Evaluation of the Metabochip Genotyping Array in African Americans and Implications for Fine Mapping of GWAS-Identified Loci: The PAGE Study
Source: PLoS One. 2012 Apr 23;7(4):e35651. doi: 10.1371/journal.pone.0035651 (PMC3335090; doi:10.1371/journal.pone.0035651)
Supplement: Table S4 — Metabochip SNP overall pass rate by minor allele frequency in the PAGE African American sample. Intervals are open on the left and closed on the right. The overall pass rate, including monomorphic SNPs, was 88.7%. (DOCX) [file pone.0035651.s005.docx]

|  |  |
| --- | --- |
| MAF Range | Overall Pass Rate (%) |
|  |  |
| 0% | 88.7 |
| (0.0%, 0.1%] | 85.0 |
| (0.1%, 1.0%] | 87.0 |
| (1.0%, 2.5%] | 92.3 |
| (2.5%, 5.0%] | 91.3 |
| (5.0%, 10.0%] | 89.5 |
| (10.0%, 50.0%] | 88.6 |
|  |  |
|  |  |

**Supporting Information Table S4:** Metabochip SNP overall pass rate by minor allele frequency in the PAGE African American sample. Intervals are open on the left and closed on the right. The overall pass rate, including monomorphic SNPs, was 88.7%.
